# Dennett’s Dental Naboli

**Published:** 1880-07

**Authors:** 


### DENNETT'S DENTAL NÄBOLÏ—WHAT IS IT?

Recently a circular with a "little book" came to hand bearing the above name, and immediately the question, "Is it a fraud?" came to mind; and almost every one does and will ask the same question upon first seeing the *circular book.*

The name bears the ear marks of quackery, *Naboli,* does one in a thousand of the profession know what the word means? It is a name in the dark. It conveys no idea to the profession, and so Mr. Dennett says in his little book, "Naboli is a liquid, and not a filling." How very condesending in Mr. D. to tell us that; otherwise we might have thought it something to kill potatoe bngs, or remove corns. How apt the antithesis between a fluid and a filling. The thing is a fluid, it is not zinc nor any of its preparations, it is not lead, it is not *oil of creosote,* it is not phosphorus, it is not arsenic, &c., but it is a fluid nevertheless, and that is as near as we can get to it by this little book. Another ear-mark, unmistakable, is a "*capital stock of* $1,000,000 fully paid up." Up where? Wouldn't it be well to add eight or nine more ciphers all paid *up*, and make it a big thing at once? Well, really, isn't this a good deal of powder for the game. A thousand pound Parrott Gun to shoot a *June bug.* Another ear-mark, "$20 a year for an office license including one dental chair; and $10 a year for each and every extra dental chair, payable annually in advance." It is not impossible that a statement of this kind might be made of something valuable, but such are exceedingly rare, if to be found at all.

The array and character of testimonials constitute another ear-mark of quackery. These are from judges, ministers, lawyers, professors, ex-governors, violinists, elocutionists, agents of oil companies, physicians, of distinction too, Mrs. So-and-So, Miss So-and-So, and plain Mr. So-and So. Oh, yes, and the president of the Massachusetts Dental Society was induced to give a certificate to the value of *Naboli.* This he did April 26, 1880, but in about a month wrote the following letter of recantation given below in the proceedings of the Massachusetts Dental Society on the subject.

I have made this presentation of this matter to answer numerous enquiries about it. If a body is to be humbugged it is well they should have their eyes open.

The array of testimonials presented I regard as the strongest proofs of charlatanry that attach to it.

The following is from the proceedings of the Massachuetts Dental Society in reference to this matter:

"At the meeting of the Massachusetts Dental Society last Friday, the undersigned were appointed a committee to acquaint the profession throughout the country with the worthlessness of the claims of the "Dennett Dental Naboli Company" to be the owners of a grand discovery for painless dentistry, and of the nature of the compound they are licensing dentists to use. "Naboli" has been analyized by able chemists, one of whom gives the following certificate:

MASSACHUSETTS COLLEGE OF PHARMACY,
CHEMICAL LABORATORY,
BOSTON, June 2, 1880.

I hereby testify that I have tested the specimens of "Naboli" for sale by the "Dennett Dental Naboli Company," which have been submitted to me, and find it to be a sweet oil, glycerate of tannin and chloroform, all scented with rose.

B. F. DAVENPORT, M. D.
Adj-Prof. of Analytical Chemistry.

The analyses show that the materials of which "Naboli" is composed have long been in use in dentistry for the same purpose. In proof of which see "Harris' Dental Dictionary," edition 1855, which says under the heading Tannic Acid. "It is a powerful astringent, and the use of it has been recommended for allaying the sensibility of sensitive teeth, preparatory for their preparation for filling;" also, "Taft's Operative Dentistry," page 241, edition 1868, which mentions the use of tannic acid and glycerine for obtunding sensitive dentine; also the Dental Times, vol. 5, page 15, 1867, which speaks of the use of chloroform and tannic acid for relieving sensibility of dentine; and in many other publications. It is learned from the state archives at Hartford that this company which claims a "capital stock of $1,000,000 fully paid up and forever unassessable," had at the date of its organization only a copyright for a circular, no patent upon the compound called "Naboli" has, up to the present date, been issued. The company claim that a patent has been allowed; but until a patent is issued, it has no force to prevent the free use of the material which it aims to control. It is believed that no patent will ever be issued for "Naboli" for the reason as shown above, that it has long been public property. Many of the dentists who are advertised as licensees were given their licenses for the sake of their names. The following from Prof. Chandler, will explain itself:

BOSTON, June 5, 1880.

I hereby certify that the license to use "Naboli" was given me by the company and was signed by me with the understanding on my part that I was to investigate the material and report upon it to the profession. The use of my name in the advertisements of the company is entirely unauthorized, and gives a false impression. I disapprove of such methods of transacting business and have therefore forbidden the further use of my name for that or any other purpose. THOMAS H. CHANDLER.

Many of those whose testimonials, or names as references, are published in the pamphlet of the company, were stockholders on record May 5th, 1880, and interested in the success of the company. Among these are Dr. Charles G. Davis, 1,000 shares; the wife of Prof. J. Jay Watson, 2,000; Dr. C. J. Blake, 400 shares; Judge C. W. Stanley, 8 shares; Ole Bull, 5 shares, and many others, having from 5 shares and upward. The resolutions published in the HERALD of Friday denouncing the company as a stock jobbing concern, designed to impose upon the public and blackmail the profession, were passed unanimously. As an offset to the recent publication in the Boston papers of the old testimonial, copied from their pamphlet, of the president of the Massachusetts Dental Society, we append the following letter, in which he apologizes to the society, and reinstates himself in the respect of honest men, by a manly though humiliating confession of his wrong. For this manly course, the company in revenge, published his old testimonial, and thus oblige us to publish his confession and recantation.

BOSTON, June 4, 1880.

I sincerely regret that while holding the position of president of the Massachusetts Dental Society I violated its by-laws by giving a certificate, dated April 26, 1880, in favor of "Naboli,' and hereby apologize to the society therefore. I acknowledge I made a great mistake in aiding the company in establishing its claims while I was pecuniarly interested, as a stockholder, in the success of the company. In view of my communication of an obtundent to Dr. Dennett, early in 1879, and of what I now know of the matter, I do not think the credit given in my certificate to H. E. Dennett, as a discoverer, is deserved.

C. G. DAVIS.

In conclusion we would state that it is considered entirely unnecessary for a dentist to have a license to use such old and well-known remedies, and we must regard the stock of this company as utterly worthless.

L. D. SHEPARD,

A. M. DUDLEY, } Committee.

